# Ergot of Wheat

**Published:** 1857-01

**Authors:** 


*Ergot of Wheat.*—Dr. Robert makes the following statements respecting this substance: 1. The medical and obstetrical property of this ergot is as incontestable as of ergot of rye, and its effects are as prompt, as direct, and as great. 2. Its hæmostatic action appears certain. Dr. Robert has administered it several times against abundant discharges of blood, and immediately after labor it has almost constantly and fully succeeded. 3. In the dose of one or two grammes, according to urgency, it has frequently succeeded in lessening, if not in completely arresting the hemorrhage; and this without appearing to produce any stimulant action on the uterus.—*Gaz. des Hopitaux, March,* 1855.

